# Magneto-Responsive Networks Filled with Polydopamine and Silane Coupling Agent Dual-Modified Carbonyl Iron Particles for Soft Actuators

**DOI:** 10.3390/polym17162228

**Published:** 2025-08-15

**Authors:** Xiushang Du, Zhenjie Zhao, Xuhang Zhang, Jingyi Zhu, Yingdan Liu

**Affiliations:** Center for Advanced Structural Materials, State Key Laboratory of Metastable Materials Science and Technology, College of Materials Science and Engineering, Yanshan University, Qinhuangdao 066004, China; dxs03416@163.com (X.D.); zhenjie_shine@163.com (Z.Z.); zhxuhang@163.com (X.Z.)

**Keywords:** magnetorheological elastomer, carbonyl iron powder, surface modification, elastic modulus, soft actuator

## Abstract

Magnetorheological elastomers (MREs) are a type of smart materials formed by dispersing magneto-responsive micron particles in an elastic polymer matrix. They hold significant potential for various applications due to their tunable stiffness, capability to carry out non-contact actuation, and rapid responsiveness to magnetic fields. However, weak interfacial interactions and poor dispersion of magnetic particles within the polymer matrix often lead to diminished magnetorheological (MR) performance. In this study, carbonyl iron powder (CIP) was chemically modified via polydopamine (PDA) deposition followed by grafting with isobutyl (trimethoxy)silane (IBTMO) to enhance its compatibility with a silicone-based matrix. The resulting anisotropic MREs fabricated using the dual-modified CIP exhibited a reduced elastic modulus, enhanced elongation, a large magnetically induced bending angle of 38°, and a notably improved MR effect of 246.8%. Furthermore, a magnetic soft actuator was designed based on the anisotropic dual-modified CIP-based MRE. When used as flippers for a duck model, the actuator successfully propelled a load approximately 76.8 times its own weight at a speed of 3.48 mm/s, thereby demonstrating promising potential for applications requiring load-bearing actuation.

## 1. Introduction

Magneto-responsive soft materials, including magnetorheological elastomers (MREs), constitute a category of smart material that allows for the real-time adjustment of their dynamic mechanical properties, like storage modulus, when exposed to an external magnetic field [1]. Owing to their rapid and reversible magneto-responsive behavior, non-contact energy transfer capabilities, precise controllability, and simplified structural design, MREs demonstrate considerable potential for applications in vibration damping [2,3,4], sensing technologies [5,6], and soft actuation systems [7,8,9]. Recently, there has been a significant increase in interest regarding the design of materials and devices based on MREs, particularly in the area of soft robotics [10]. This surge is primarily attributed to the unique magneto-responsive properties and biocompatibility of MREs [11]. Furthermore, the impact of magnetic fields on material characteristics extends beyond mechanical behavior and can also alter electrical conductivity and optical properties [12], indicating the critical role played by magnetic fields in modulating material performance.

Typically, MREs are composed of a soft elastic matrix, such as silicone rubber [5,6], natural rubber [13,14], or polyurethane [15,16], filled with magnetically responsive particles including carbonyl iron powder (CIP) [15], Fe_3_O_4_ [17,18]_,_ and NdFeB [19]. Among these fillers, CIP is particularly favored because of its high saturation magnetization and low coercivity, both of which are crucial for achieving a strong magnetorheological (MR) effect. MREs can be categorized into isotropic and anisotropic types based on the fabrication conditions. Isotropic MREs are produced in the absence of a magnetic field, resulting in a random distribution of magnetic particles within the matrix. In contrast, anisotropic MREs are fabricated under an external magnetic field, which induces the alignment of magnetic particles into chain-like structures oriented parallel to the direction of the applied magnetic field [20,21]. Notably, anisotropic MREs generally exhibit a much stronger MR effect compared to isotropic ones due to the pre-formed chain structure which is favorable for higher magnetization and interactions among particles [22,23]. In addition to the arrangement of the magnetic particles, the interfacial interactions between the magnetic particles and the polymer matrix play a pivotal role in determining both the mechanical properties and MR performance of MREs [24,25,26]. The inherent incompatibility between inorganic magnetic particles and organic elastomeric matrices often results in weak interfacial adhesion, leading to issues like particle agglomeration, interfacial debonding, and inefficient stress transfer. These phenomena ultimately limit the mechanical properties, MR effect, and practical applications of MREs [27].

Recent studies have demonstrated that the surface modification of magnetic particles can effectively enhance their dispersion and interfacial compatibility, leading to improved MR properties of MREs. For example, applying polystyrene coatings on CIP reduced the initial storage modulus and enhanced the MR effect by 28.04% [28]. Additionally, the modification of CIP with poly (trimethylsilyloxyethyl methacrylate) enhanced their compatibility with the silicone rubber matrix via physical entanglement, resulting in an increase in the MR effect from 42.8% to 66% [29]. Similarly, CIP modified with vingyltriethoxysilane exhibited a 37% increase in the MR effect due to the stronger interfacial interactions with the silicone rubber matrix [30]. Therefore, the application of silane coupling agents for surface modification has been demonstrated to effectively enhance the MR effect. However, the Fe-O-Si-C bonds formed during this modification process are prone to hydrolysis in aqueous environments, potentially compromising the mechanical properties and long-term stability of the MREs [31]. It has been demonstrated that constructing a bridging layer using polydopamine (PDA) was an effective way of inhibiting the hydrolytic degradation of the coating [32]. Additionally, surface modification greatly affects the elastic modulus of composite elastomers, which is essential for the deformation ability of MREs when used as soft actuators. Therefore, it is important to develop an effective surface modification method that can enhance the compatibility between CIP and the rubber matrix, thereby improving the MR effect of the MREs while mitigating potential issues.

Herein, anisotropic MREs based on silicone rubber with a superior MR effect were fabricated through the incorporation of dual-modified CIP with PDA and a silane coupling agent (isobutyl (trimethoxy)silane, IBTMO). Specifically, PDA creates a robust adhesive layer on the surface of the CIP, promoting the attachment of IBTMO and preventing the hydrolysis of Fe-O-Si-C bonds. The novel dual modification of CIP results in a hydrophobic interface that enhances the dispersion of CIP and their compatibility with the matrix. The modifications are anticipated to improve not only the mechanical performance but also the MR effect significantly. This research thoroughly examines the magnetic characteristics, surface chemistry, and morphology of the modified CIP. Both isotropic and anisotropic MREs were prepared using modified and unmodified CIPs. Furthermore, this study analyzes the microstructure, mechanical properties, magnetic field-induced variation in viscoelasticity, and bending performance of the MREs with either type of CIPs. Ultimately, a magnetic soft actuator was developed using the anisotropic MRE containing dual-modified CIP. In a typical application situation, it can propel a rubber toy duck across the surface of water in a manner akin to the movement of flippers, demonstrating the significant potential of this developed MRE based on the dual-modified CIP for use in load-bearing soft actuation applications.

## 2. Materials and Methods

### 2.1. Materials

The polydimethylsiloxane (PDMS) precursor and curing agent (Sylgard 184) were purchased from Dow Corning. Dopamine hydrochloride (DA⋅HCl) and CIP (CN type, average particle size ~3 µm) were obtained from Macklin. Tris (hydroxymethyl)aminomethane (Tris) was supplied by TCI (Shanghai, China). Isobutyl (trimethoxy)silane (IBTMO) was purchased from Aladdin (Shanghai, China).

### 2.2. Surface Modification of CIP

CIP was ultrasonically treated in ethanol for 25 min to remove surface impurities and enhance surface activity [32], followed by triple rinsing with deionized water. An aqueous DA solution with a concentration of 2 g/L was prepared and Tris was added to adjust the pH of the DA solution to 8.5. Then, the cleaned CIP was dispersed in 300 mL of DA solution and stirred mechanically at 50 °C for 4 h to allow for PDA coating. Subsequently, IBTMO was added to the mixture at a mass ratio of IBTMO to DA of 10:1, and the reaction proceeded at 70 °C for 6 h. The dual-modified CIP was collected by centrifugation and dried at 50 °C. The surface-modified product is referred to as DCIP.

### 2.3. Fabrication of MREs

**Figure 1:**
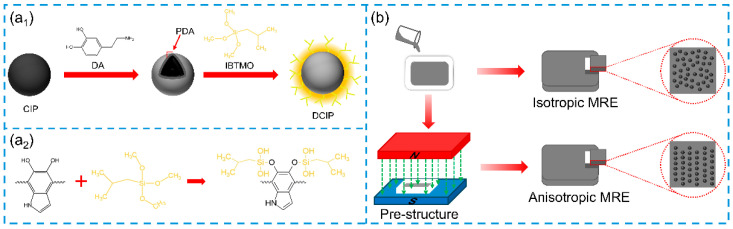
Schematic diagram of (**a_1_**) the surface modification of CIP by PDA and IBTMO, (**a_2_**) the chemical reaction between PDA and IBTMO, and (**b**) the preparation procedure of isotropic and anisotropic MREs.

First, the PDMS precursor and curing agent were mixed at a mass ratio of 20:1. Isotropic and anisotropic MREs were fabricated by incorporating 60 wt% of either CIP or DCIP into the mixed solution. For isotropic MREs, the CIP was then added to the mixture and stirred thoroughly with a glass rod for 10 min. The resulting mixture was poured into a mold, degassed under vacuum for 15 min to eliminate air bubbles, and subsequently cured at 80 °C for 4 h. To prepare anisotropic composites, the uncured mixtures were placed in a uniform magnetic field of 30 mT during the curing process to align the magnetic particles. The produced composites are referred to as iso-CIP, iso-DCIP, ansio-CIP, and ansio-DCIP according to the type of incorporated particles and whether the magnetic field was or was not applied during the fabrication process. The surface modification procedure of CIP as well as the fabrication process of the MREs are illustrated in Figure 1.

### 2.4. Characterization

The magnetic properties of CIP and DCIP particles were characterized at ambient temperature utilizing a physical property measurement system (PPMS 7404, Lake Shore, Columbus, OH, USA). Scanning electron microscopy (SEM) (Sigma 360, Zeiss, Oberkochen, Germany) was employed to examine the morphologies of the CIP and DCIP particles, as well as the internal microstructures of the MREs. Elemental mapping analysis was conducted using energy-dispersive spectroscopy (EDS) to visualize the distribution of characteristic elements on the surface of the modified particles and to assess the dispersion of iron (Fe) within the composite matrices. The thermal stability of CIP and DCIP particles was evaluated through thermogravimetric analysis (TGA) (STA449-F5, Netzsch, Bavaria, Germany). The measurements were conducted over a temperature range of 25 to 800 °C, with a heating rate of 10 K/min, under an argon atmosphere.

The tensile mechanical properties of the MREs were evaluated utilizing a universal tensile testing machine (TFW-5S, Tuofeng, Shanghai, China). Dumbbell-shaped specimens with dimensions of 50 mm × 4 mm × 1 mm were tested at a tensile rate of 50 mm/min under ambient conditions. For anisotropic samples, the tensile load was applied orthogonally to the orientation of the aligned particle chains. Each test was conducted at least three times, and the mean values for the elastic modulus and elongation at break were documented. The dynamic rheological behavior of the MREs was examined using a rotational rheometer (MCR502, Anton Paar, Graz, Austria) equipped with an MR device (MRD70/1T). Shear oscillatory tests were conducted at 25 °C under various magnetic fields ranging from 0 to 1 T, using disk-shaped samples with a diameter of 20 mm and a thickness of 1 mm. The magnetic field was applied perpendicularly to the surface of the MRE sample.

A custom-built magnetic actuation system was employed to assess the magnetically responsive motion behavior of the MREs. The setup included a DC power supply (SPPS-C305, Kuaiqu, Shenzhen, China), an electromagnet (LSD-P120/70, Langshuo, Wenzhou, China), and a teslameter (HT20, Hengtong, Shanghai, China). The system enabled biomimetic linear swimming of magnetic soft robots, and motion was recorded using a high-resolution camera.

## 3. Results and Discussion

### 3.1. Morphology, Chemical Composition, and Magnetism of Surface-Modified CIP

**Figure 2:**
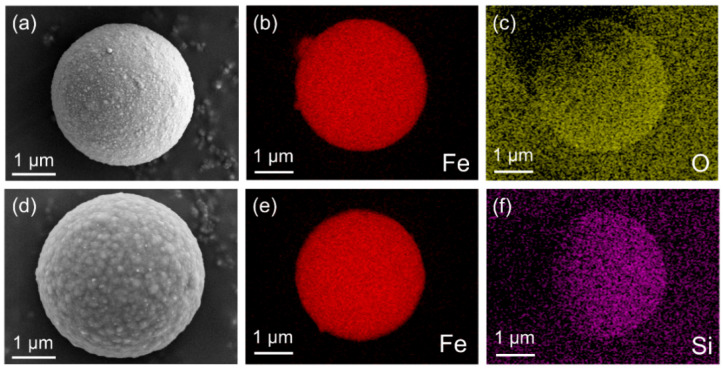
SEM and EDS images of (**a**–**c**) CIP and (**d**–**f**) DCIP.

The microstructure of the CIP and DCIP was examined using SEM, as shown in Figure 2 and Figure S1. The CIP particles are spherically shaped with a diameter range of 3–4 m. The pristine CIP (Figure 2a) exhibits a rough surface morphology, whereas the DCIP (Figure 2d) modified by PDA and IBTMO displays a smoother surface with uniformly distributed bumps in hundreds of nanometers because of the polymerization of DA, indicating successful surface modification. EDS analysis further confirms the presence of nitrogen (Figure S1d) and silicon (Figure 2f), a characteristic element of the PDA and silane coupling agent IBTMO, respectively, verifying the effective grafting of IBTMO onto the particle’s surface.

**Figure 3:**
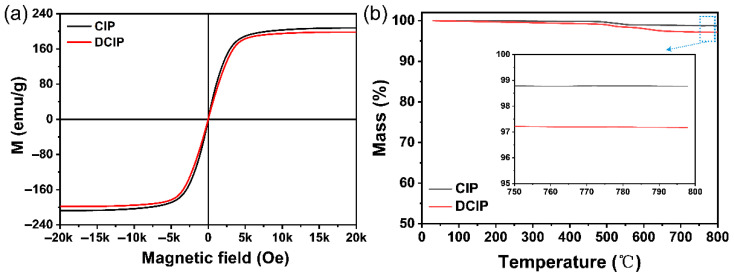
(**a**) Magnetic hysteresis loops and (**b**) TGA curves of CIP and DCIP particles.

The magnetic hysteresis loops of CIP and DCIP particles are displayed in Figure 3a. Both samples exhibit zero coercivity and negligible hysteresis, indicating typical soft magnetic behavior with low coercivity [32]. The saturation magnetization values of CIP and DCIP are 207.6 emu/g and 198.1 emu/g, respectively, suggesting that the surface modification exerts a negligible effect on the intrinsic magnetic properties of CIP. The TGA curves of CIP and DCIP particles under an argon atmosphere are displayed in Figure 3b. Compared to CIP, the DCIP particles exhibit a higher mass loss of 2.9% at 800 °C, while CIP alone shows a mass loss of only 1.2%. The increased weight reduction is ascribed to the decomposition of the PDA@IBTMO coating layer.

### 3.2. Microstructures and Mechanical Properties of the MREs

**Figure 4:**
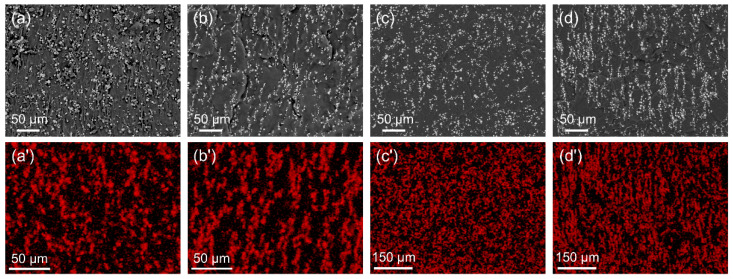
SEM images of the MREs of (**a**) iso-CIP, (**b**) aniso-CIP, (**c**) iso-DCIP, and (**d**) aniso-DCIP; EDS mapping of Fe element for the MREs of (**a’**) iso-CIP, (**b’**) aniso-CIP, (**c’**) iso-DCIP, and (**d’**) aniso-DCIP.

Figure 4a–d present the SEM images of both isotropic and anisotropic MREs, while the corresponding EDS elemental mappings of iron (Fe) are presented in Figure 4a’–d’. In the isotropic MREs, CIP and DCIP particles are randomly distributed within the silicone rubber matrix, with the noticeable aggregation of particles, particularly in the MREs containing CIP. In contrast, the anisotropic MREs display a pronounced alignment of particles parallel to the direction of the applied magnetic field, resulting in distinct chain-like structures. This alignment demonstrates the effective orientation of magnetic particles under the external magnetic field, which contributes to the anisotropic nature of the MREs.

**Figure 5:**
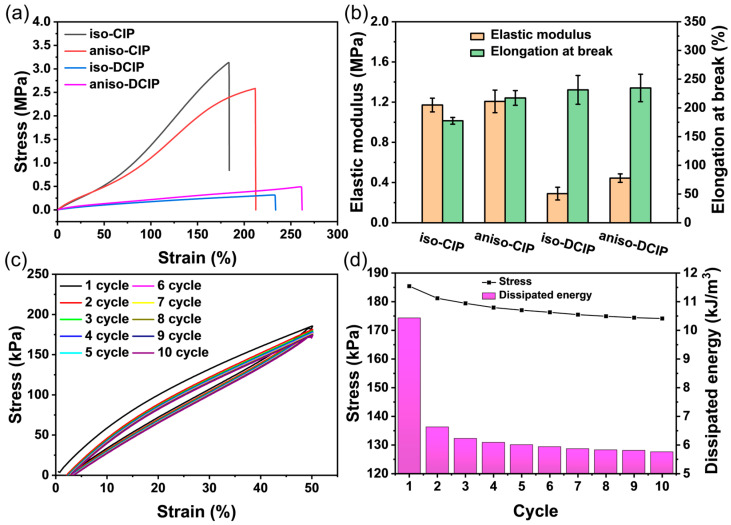
(**a**) Stress–strain curves; (**b**) elastic modulus and elongation at break for both isotropic and anisotropic MREs; (**c**) tensile curves for 10 consecutive cycles at a fixed strain of 50% and (**d**) the corresponding stress and dissipated energy.

The representative stress–strain curves of isotropic and anisotropic MREs are displayed in Figure 5a, while Figure 5b summarizes their elastic modulus and elongation at break. The MREs based on DCIP particles showed much lower tensile stress but improved elongation at break. Specifically, the elongation at break of aniso-CIP increases from 212.43% to 262.02% upon surface modification. This enhancement is primarily ascribed to the enhanced dispersion of DCIP particles within the silicone matrix, as well as the strengthened interfacial interactions between the particles and the polymer network. Comparable findings were reported for MREs containing PDA and dodecyltrimethoxysilane-modified CIP, further confirming the role played by surface modification in improving the dispersion and interfacial interaction [32]. As shown in Figure 5b, the elastic modulus of the DCIP-based MREs is markedly lower than that of CIP-based MREs, which is only 0.44 MPa for aniso-DCIP and 1.21 MPa for aniso-CIP. The lower elastic modulus of aniso-DCIP indicates that it is much softer than aniso-CIP. Furthermore, the DCIP-based MREs demonstrate considerably improved elongation at break compared to the CIP-based MREs. The aniso-DCIP MRE exhibited excellent elastic recovery and fatigue resistance, as evidenced by the large overlap of successive loading cycles and the progressive stabilization of dissipated energy from the second to the tenth cycle, as depicted in Figure 5c,d. These findings substantiate the long-term stability of the MREs.

### 3.3. Magnetorheological Properties of the MREs

**Figure 6:**
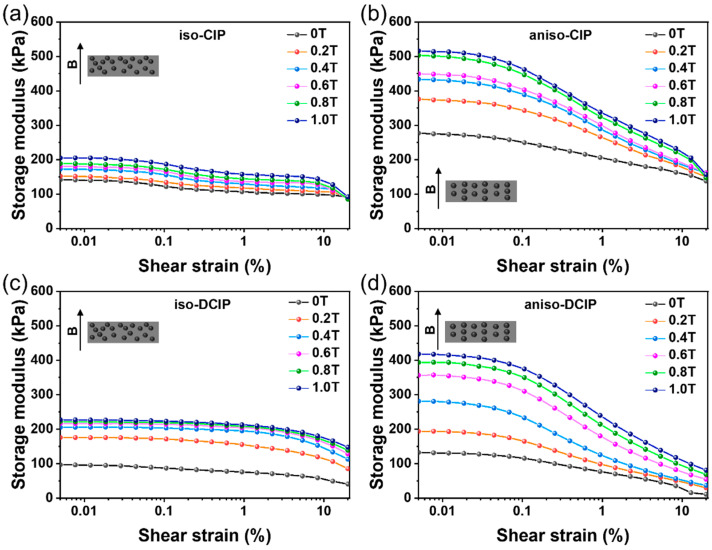
Dependence of storage modulus on strain amplitude under different magnetic fields for the MREs of (**a**) iso-CIP, (**b**) aniso-CIP, (**c**) iso-DCIP, and (**d**) aniso-DCIP. The inset illustrates the direction of the applied magnetic field.

The dynamic viscoelastic properties of the prepared MREs were first examined in strain sweeps to identify the linear viscoelastic region of the MREs. The strain sweep tests were conducted at a constant frequency of 10 Hz with strain amplitudes ranging from 0.005% to 20%. The dependence of the storage modulus on oscillatory shear strain for isotropic and anisotropic MREs is shown in Figure 6a–d, respectively. All samples exhibit typical viscoelastic behavior: the storage modulus stays nearly constant at low strains but decreases markedly at higher strains, indicative of the Payne effect [33]. This effect becomes more pronounced under stronger magnetic fields due to enhanced interactions among the particles, resulting in increasingly weakened storage modulus as strain increases [20]. Furthermore, the anisotropic MREs exhibit a more rapid decrease in storage modulus under high shear strain compared to the isotropic ones. This behavior is primarily attributed to the disruption of the chain-like structure of magnetic particles by high shear strain [32].

**Figure 7:**
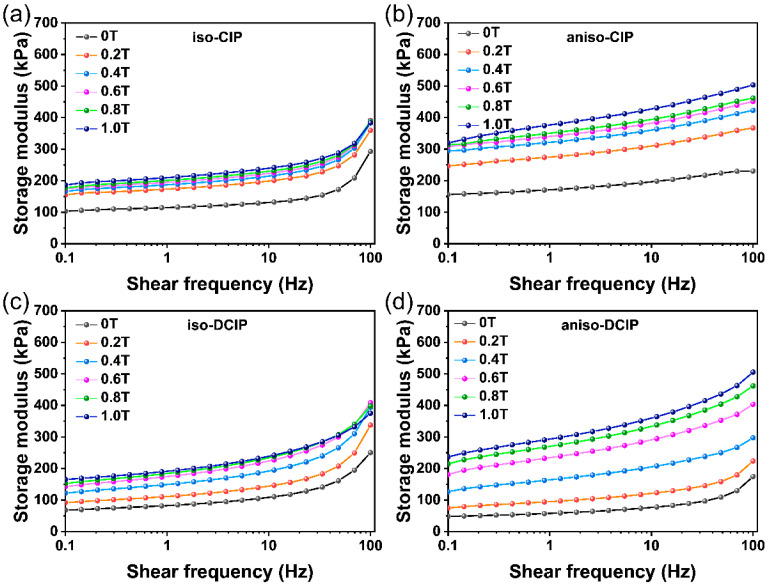
Dependence of storage modulus on shear frequency under different magnetic fields for the MREs of (**a**) iso-CIP, (**b**) aniso-CIP, (**c**) iso-DCIP, and (**d**) aniso-DCIP.

Following the strain sweep tests, the viscoelastic behavior of the MREs in frequency sweep was evaluated. The strain amplitude was fixed at 0.05%, while the frequency varied from 0.1 to 100 Hz. Figure 7a–d display the dynamic storage modulus of the MREs at various magnetic fields. The storage modulus of the MREs containing CIP and DCIP in the dynamic state increases with frequency at a specific magnetic field [29]. This phenomenon is attributed to a shorter response time at higher frequencies, which leads to a phase lag between the deformation of the silicone rubber molecular chains and the applied shear force, resulting in greater dynamic stiffness in the MREs during the oscillatory testing. In addition, it was noted that all MREs exhibited a higher storage modulus as the magnetic field rose.

**Figure 8:**
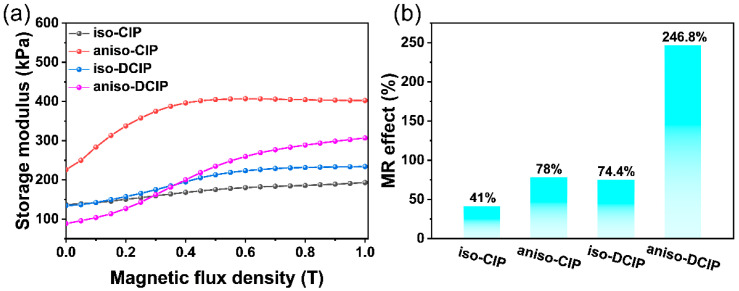
(**a**) Dependence of shear storage modulus on magnetic field and (**b**) relative MR effect for the MREs of iso-CIP, aniso-CIP, iso-DCIP, and aniso-DCIP at the magnetic field of 1 T.

The storage modulus of MREs as a function of magnetic field is shown in Figure 8a, measured at a fixed strain amplitude of 0.05% and a frequency of 10 Hz, with the magnetic field strength ranging from 0 to 1 T. For both isotropic and anisotropic MREs, the storage modulus increases with the magnetic field, reflecting enhanced particle magnetization and stronger interactions among the magnetic particles. Notably, the DCIP-based MREs exhibit a lower zero-field storage modulus compared to CIP-based MREs, attributed to improved particle dispersion. The relative MR effect is defined as follows:

(1) $$M=\frac{G^{\prime }-G_{0}^{\prime }}{G_{0}^{\prime }}\times 100\%$$

where *M* denote the relative MR effect and *G′* and *G′_0_* denote the storage modulus of the MRE measured at a specific magnetic field and zero-field, respectively. As shown in Figure 8b, the DCIP-based MREs exhibit significantly higher MR effects compared to CIP-based MREs. Specifically, the MRE of aniso-DCIP achieves an MR effect of 246.8%, which is 216.4% greater than the MRE of aniso-CIP. The relative enhancement is 81.5% for iso-DCIP compared with iso-CIP. Furthermore, the anisotropic MREs of both CIP and DCIP reach MR effects of 78% and 246.8%, respectively, both surpassing their isotropic equivalents. This pronounced enhancement arises from stronger interactions among magnetic particles facilitated by the chain-like alignment of particles under the applied magnetic field, leading to increased interparticle attraction and shear modulus.

### 3.4. Magnetic Field Response and Actuation Performance of the MREs

**Figure 9:**
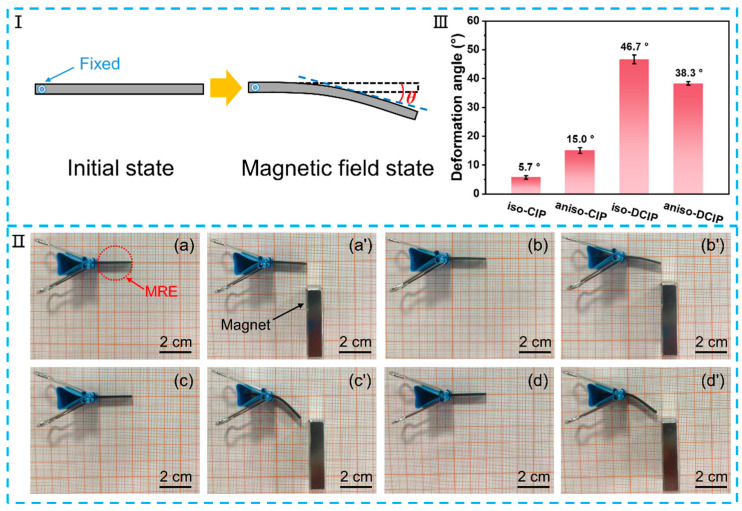
(**I**) Schematic diagram of bending process of the MRE at magnetic field. (**II**) Optical images of the bending performance of the MREs of (**a**,**a’**) iso-CIP, (**b**,**b’**) aniso-CIP, (**c**,**c’**) iso-DCIP, and (**d**,**d’**) aniso-DCIP. (**III**) The deformation angle of the MREs.

The soft MREs demonstrate bending capabilities when subjected to a gradient magnetic field, with one end secured by a clip. Figure 9I illustrates the bending process of the MREs, where they experience deformation under an external magnetic field, with θ indicating the angle of deformation. The bending performance of the MREs in a magnetic field is depicted in Figure 9II, showing that both types of MREs bend due to magnetic attraction [9,34]. The DCIP-based MREs exhibit significantly greater bending compared to those based on CIP, which may be related to their lower elastic modulus. As illustrated in Figure 9III, the bending angle of the DCIP-based MREs surpasses that of the CIP-based MREs. Notably, iso-DCIP reaches the largest bending angle of 46.7°, which is much higher than 5.7° shown by iso-CIP. aniso-DCIP has a bending angle of 38.3°, slightly lower than that of iso-DCIP, but also significantly higher than CIP-based MREs. This enhanced bending performance is attributed to the organic coating on the CIP, which improves the compatibility between the particle and the matrix, resulting in a softer composite elastomer and a larger bending angle under identical magnetic field conditions. In addition, the bending angle of iso-DCIP is 8.4° greater than that of aniso-DCIP, which is attributed to the softer characteristic or lower elastic modulus of the iso-DCIP MRE, as shown in Figure 5b, which means that the bending angle is mostly dependent on the stiffness of the MREs rather than the MR effect.

**Figure 10:**
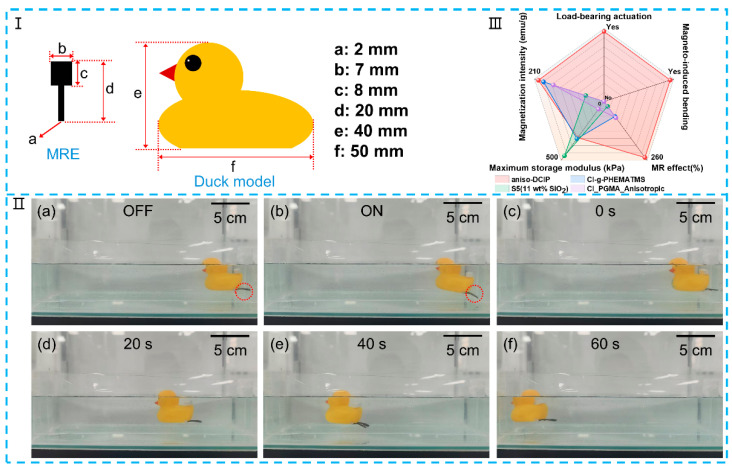
(**I**) Schematic diagram of duck model using MRE actuator as its flippers. (**II**) Optical images of a “swimming duck” based on the actuation of the MRE: (**a**,**b**) with magnetic field “off” and “on”; (**c**–**f**) change in displacement over time. (**III**) Radar chart comparing the integrated performance of four MREs, aniso-DCIP, CI-g-PHEMATMS [29], S5 (11 wt% SiO_2_) [35], and CI_PGMA_Anisotropic [36], across load-bearing actuation, magneto-induced bending, MR effect, maximum storage modulus, and magnetization intensity.

A magnetic soft actuator was designed using the MRE of aniso-DCIP due to its premium MR effect and relatively large deformation angle. The MRE is cut into the shape as shown in Figure 10I, with a total weight of 0.45 g (two pieces). When adhered onto the end bottom of a rubber duck model, the soft actuator performs repetitive “bending–straightening” operations controlled by an electromagnet. Upon application of an external magnetic field, the soft actuator bends; once the field is removed, it recovers to its original shape, generating a driving force that propels the duck (weighing 34.56 g) forward in water. Figure 10(IIa,b) illustrate the actuator’s deformation under a switched magnetic field. The actuator successfully overcomes water resistance and undergoes bending. The displacement of the rubber duck over time is shown in Figure 10(IIc–f), demonstrating that it advances 209 mm within 60 s under a magnetic induction of 30 mT. This corresponds to an average velocity of 3.48 mm/s over a 60 s interval, demonstrating effective magnetic actuation. This indicates that the actuator possesses strong application potential for load-bearing actuation. The MRE of aniso-DCIP has excellent comprehensive performance, as shown in Figure 10III. Compared with previously reported works including the MRE of CIP particles grafted with poly (trimethylsilyloxyethyl methacrylate) chains [29], the MRE prepared with silica nanoparticles as an additive [35], and the MRE of CIP particles encapsulated with a poly (glycidyl methacrylate) shell [36], the MRE of aniso-DCIP not only shows distinctive magneto-induced bending performance and load-bearing actuation capability but also presents a higher storage modulus at magnetic field than that of CI_PGMA_Anisotropic [36] and a much higher MR effect of 246.8%.

## 4. Conclusions

In this study, the magnetic CIP was dual-modified by PDA and IBTMO to enhance the interfacial interaction with the silicone rubber matrix. This surface modification improved the compatibility between CIP and the matrix, leading to a significant increase in the elongation at break of the MREs. As a result of a more efficient magnetic interaction transfer within the matrix, the relative MR effect of isotropic and anisotropic MREs based on dual-modified CIP was enhanced by 81.5% and 216.4%, respectively. Furthermore, a magneto-responsive soft actuator was developed using the MRE of aniso-CIP. Mimicking the flapping motion of flippers, the actuator bends under an external magnetic field, enabling the soft robot to achieve forward locomotion at an average speed of 3.48 mm/s. This easily fabricated soft actuator achieves the desired actuation performance, indicating the significant potential of aniso-DCIP for applications in biomedical and bionic systems.

## Data Availability

The data presented in this study are available upon request from the corresponding author.

## Supplementary Materials

The following supporting information can be downloaded at: https://www.mdpi.com/article/10.3390/polym17162228/s1. Figure S1: The EDS electronic images of (a) CIP and (b) DCIP; the EDS elemental images of (c) oxygen and (d) nitrogen in DCIP; Video S1: The movement of the actuator in a single switching magnetic field; Video S2: The displacement of the actuator varies over time under a continuous switching magnetic field.
