# Supplementary material for: Magneto-Responsive Networks Filled with Polydopamine and Silane Coupling Agent Dual-Modified Carbonyl Iron Particles for Soft Actuators
Source: Polymers (Basel). 2025 Aug 15;17(16):2228. doi: 10.3390/polym17162228 (PMC12390496; doi:10.3390/polym17162228)
Supplement: Supplementary file 1 [file polymers-17-02228-s001.zip › Supporting Information.pdf]

supplementary

Magneto-Responsive Networks Filled with Polydopamine and Silane Coupling Agent Dual-Modified Carbonyl Iron Particles for Soft Actuators

Xiushang Du, Zhenjie Zhao, Xuhang Zhang, Jingyi Zhu* and Yingdan Liu*

Center for Advanced Structural Materials, State Key Laboratory of Metastable Materials Science and Technology, College of Materials Science and Engineering, Yanshan University, Qinhuangdao 066004, China

Corresponding author email: jyzhu@ysu.edu.cn (J.Z.); ydliu@ysu.edu.cn (Y.L.)

Supplementary Figures

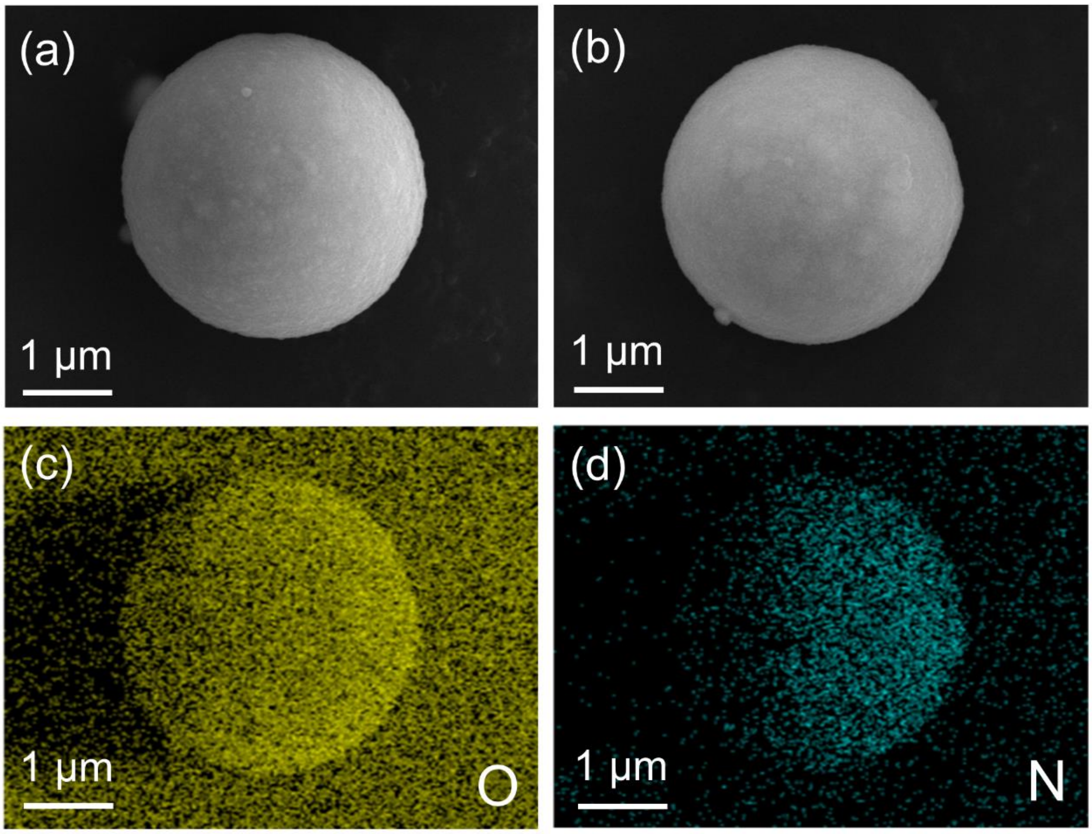

Figure. S1: The EDS electronic images of (a) CIP and (b) DCIP; The EDS elemental images of (c) oxygen and (d) nitrogen in DCIP.

Supplementary Video

Video S1: The movement of the actuator in a single switching magnetic field

Video S2: The displacement of the actuator varies over time in a continuous switching magnetic field
